# Exploring differences among serial, portfolio, and novice entrepreneurs: A study of demographic and psychological factors in micro and small enterprises

**DOI:** 10.1371/journal.pone.0332523

**Published:** 2025-09-19

**Authors:** Jarosław Ropęga, Agnieszka Lipińska-Grobelny

**Affiliations:** 1 Department of Entrepreneurship and Industrial Policy, Faculty of Management, University of Lodz, Lodz, Poland; 2 Department of Work Psychology, Organization and Career Counseling, Institute of Psychology, University of Lodz, Lodz, Poland; West Pomeranian University of Technology, POLAND

## Abstract

The aim of this study was to empirically validate selected demographic and psychological factors from Shane’s model in the context of serial and portfolio entrepreneurship. The analysis involved 326 entrepreneurs, including 110 serial entrepreneurs, 105 portfolio entrepreneurs, and 111 novice entrepreneurs (control group). The study employed a survey questionnaire combined with five validated psychological instruments, administered in a manner that enabled control for common method bias. The analysis revealed that two demographic factors—education and having entrepreneurial parents—were statistically significant for distinguishing serial and portfolio entrepreneurs. In terms of personality traits, differences were observed in the levels of psychological capital, suchas: hopefulness, resilience, and also risk-taking propensity, conformity-nonconformity behaviour, with the portfolio entrepreneurs scoring the highest in these areas. This study successfully validates Shane’s model in relation to micro and small-scale entrepreneurship, contributing to a deeper understanding of entrepreneurial behaviour.

## Introduction

Despite growing recognition that many entrepreneurs engage in more than one venture across their careers, research into the specific characteristics of such individuals remains limited. Over the past two decades, scholars have increasingly called for a deeper understanding of entrepreneurs who launch multiple businesses – either sequentially (serial entrepreneurs) or simultaneously (portfolio entrepreneurs). As early as the 1980s, MacMillan emphasized that gaining insight into these types of entrepreneurs is critical to building a comprehensive theory of entrepreneurship [1]. Today, habitual entrepreneurs represent a substantial and influential segment of the entrepreneurial landscape.

This study seeks to address this gap by empirically testing selected demographic and psychological variables from Shane’s model of the entrepreneurial process [2]. According to Shane, opportunity recognition is a highly individualized process shaped by a person’s demographic background and psychological profile. These factors not only influence how opportunities are identified but also affect the quality of those opportunities in terms of innovation, survival, and business performance [3].

In exploring the psychological underpinnings of entrepreneurship, trait theory has long played a central role. On one hand, a number of researchers [4–8] have attempted to define entrepreneurship through a constellation of stable personality traits – such as risk-taking, resilience, and innovativeness – collectively known as the entrepreneurial personality. Trait theory offers a framework for understanding individual differences in behaviour, decision-making, and success potential. Its strength lies in its predictive utility and applicability across contexts. On the other hand, the use of trait theory has been met with substantial criticism. Early critiques argued that personality traits alone are insufficient to explain entrepreneurial outcomes. Gartner [9], for instance, found that intra-group variation among entrepreneurs often exceeds differences between entrepreneurs and non-entrepreneurs, casting doubt on the existence of a consistent entrepreneurial profile. Similarly, Kraśnicka [10] pointed out that the diversity and interaction of psychological traits produce highly individualized entrepreneurial pathways, making it difficult to construct a general model of the “typical” entrepreneur. However, more recent work by Kerr et al. [11] suggests a renewed interest in personality-based approaches, driven by improved methodologies and access to large-scale datasets. In particular, the exploration of differences between types of entrepreneurs – such as serial and portfolio – may offer a more nuanced and meaningful application of trait theory.

Building on this theoretical background, the central research question of this study is: Do serial, portfolio, and novice entrepreneurs differ systematically in the demographic and psychological characteristics identified in Shane’s model of the entrepreneurial process? Answering this question is essential not only for advancing entrepreneurship theory but also for informing policy-makers, educators, and business support organizations in designing targeted interventions that enhance entrepreneurial success across different career stages.

This study aims to contribute to this evolving discourse by examining whether serial, portfolio, and novice entrepreneurs differ in terms of key demographic and psychological characteristics identified in Shane’s model. By comparing these three groups, the research seeks to identify specific traits and background variables that distinguish habitual entrepreneurs from their novice counterparts. Ultimately, the study offers empirical support for the application of Shane’s model in the context of micro and small entrepreneurship and enhances our understanding of how different entrepreneurial paths are shaped by individual characteristics.

### Theoretical background

#### Serial, portfolio and novice entrepreneurs.

Although re-entrepreneurs are a common feature in many economies, the phenomenon of habitual entrepreneurship remains challenging to capture empirically. The proportion of entrepreneurs with previous business ownership experience varies significantly across countries, typically ranging from 30% to 50% [12]. In Poland, current data indicate that 32.44% of small entrepreneurs can be classified as re-entrepreneurs [12].

A growing body of research [13–16] highlights that prior entrepreneurial experience can have both beneficial and detrimental effects on the outcomes of subsequent ventures – particularly in micro and small enterprises, where strategic decision-making is often concentrated in the hands of a single owner-entrepreneur. Opportunity exploitation does not always involve launching a new venture; it may also involve acquiring or inheriting an existing business [17–19].

Habitual entrepreneurs are defined as individuals who currently hold or have held a minority or majority stake in two or more independent businesses, with at least one of those ventures being either founded, acquired, or inherited. This group can be further categorized into: (1) serial entrepreneurs – individuals who have exited (sold or closed) at least one business in which they held ownership and currently hold a minority or majority share in a newly established, acquired, or inherited independent business; and (2) portfolio entrepreneurs – individuals who simultaneously own minority or majority stakes in two or more independent businesses that were newly founded, acquired, or inherited [20,21]. By contrast, novice entrepreneurs are individuals without any prior business ownership experience – whether as founders, purchasers, or heirs – who currently own a minority or majority share in a single newly founded, acquired, or inherited business.

Although both serial and portfolio entrepreneurs are classified as habitual entrepreneurs, they embody fundamentally distinct entrepreneurial logics. Serial entrepreneurs tend to pursue ventures sequentially, often driven by exploration, novelty-seeking, and the opportunity to apply lessons learned from previous businesses [22]. In contrast, portfolio entrepreneurs maintain concurrent ownership of multiple ventures, balancing diversification with operational synergies. This approach requires high cognitive flexibility, tolerance for complexity, and advanced orchestration of resources [23]. Empirical evidence shows that portfolio entrepreneurs are more likely to leverage existing social capital and redeploy resources across ventures, whereas serial entrepreneurs more often undergo significant career transitions and redefine their business focus [15,24,25] These distinctions also align with the notion of bounded sustainable entrepreneurship. Serial entrepreneurs, by exiting and re-entering with new ventures, often mitigate sustainability tensions through renewal and reorientation, whereas portfolio entrepreneurs must manage these tensions concurrently across multiple businesses, balancing diversification with the psychological and organizational costs of complexity [26]

### Entrepreneurial influencing factors as the central focus of the study

Over the past two decades, researchers have increasingly highlighted the need to understand the specific profiles and dynamics of habitual entrepreneurs – those who engage in more than one business venture across their careers. These entrepreneurs, categorized as serial or portfolio form a significant segment of the entrepreneurial ecosystem [1]. Despite their growing prevalence, the underlying factors that differentiate these groups – especially in comparison to novice entrepreneurs – remain underexplored, particularly in the context of micro and small businesses where resource constraints and individual decision-making are especially salient.

Given the complexity and heterogeneity of entrepreneurial pathways, particularly among habitual entrepreneurs, it becomes crucial to examine the individual-level factors that shape their behaviour and opportunity recognition. This study draws on Shane’s model of the entrepreneurial process [2], which offers a robust theoretical foundation for analyzing how personal characteristics influence the identification and exploitation of entrepreneurial opportunities. Shane emphasizes that entrepreneurship arises from the interaction between individual traits and environmental conditions. This interaction determines both the recognition and exploitation of entrepreneurial opportunities. Opportunities are not uniformly visible to all individuals. Personal experiences, knowledge, and cognitive frameworks filter what is recognised as a viable business idea. Within this framework, both demographic factors (e.g., age, education, parental background) and psychological traits (e.g., self-efficacy, need for achievement, risk tolerance) play pivotal roles. While psychological characteristics have traditionally received more attention in entrepreneurial research, demographic variables function as important structural filters that shape access to networks, resources, and market opportunities [3].

In the context of the present study, Shane’s model serves as the conceptual bridge linking the demographic and psychological variables under investigation to the three entrepreneurial types—serial, portfolio, and novice entrepreneurs—operating within the specific conditions of small and micro-enterprises.

While Shane’s [2] model provides a valuable framework for linking individual characteristics to opportunity recognition and exploitation, its original formulation was primarily developed in the context of nascent entrepreneurs. Applying it to habitual entrepreneurs—both serial and portfolio—raises several conceptual challenges. Experienced entrepreneurs often possess entrenched cognitive schemas that shape their perception of opportunities and strategic decision-making [22]. These schemas, formed through repeated venture creation and management, enable more efficient pattern recognition but may also introduce biases, such as overgeneralisation from past successes. Recent studies suggest that cognitive frameworks for multiple venture ownership differ between serial and portfolio entrepreneurs, with the latter relying more on resource redeployment logics [23], while serial entrepreneurs tend toward strategic renewal cycles. This indicates a need to adapt Shane’s model to account for experience-driven cognition, resource path-dependency, and complex opportunity portfolios.

Despite the relevance of Shane’s model, habitual entrepreneurship has rarely been examined through this lens, particularly in relation to both serial and portfolio entrepreneurs. These two types differ not only in terms of business structure and strategy but also in how they leverage experience, manage risk, and utilize psychological resources. Serial entrepreneurs often pursue new ventures sequentially, learning from each experience and transferring knowledge over time [20]. Portfolio entrepreneurs, by contrast, manage several businesses simultaneously, requiring higher-level capabilities in multitasking, strategic planning, and resource coordination [27,28]. Those starting a business for the first time (novice entrepreneurs) serve as a useful comparison group. Unlike habitual entrepreneurs, they typically have limited experience, networks, and psychological conditioning for opportunity exploitation. Including novice entrepreneurs in the study enables a more nuanced understanding of what differentiates those who engage in entrepreneurship repeatedly from those who do so only once. Moreover, recent studies stress that psychological capital should not be seen as a static trait but rather as a dynamic and context-dependent resource. Entrepreneurial engagement over time can either reinforce or deplete psychological reserves, depending on individual coping mechanisms and organizational conditions [29]. This perspective deepens trait theory by recognizing that traits and psychological resources evolve through entrepreneurial practice.

This research was conducted within micro and small enterprises, which are typically characterized by limited capital, localized markets, and a high level of owner involvement. Although definitions vary across countries and institutions, this study aligns with the European Commission’s criteria (Recommendation 2003/361/EC) for micro and small enterprises. According to these criteria, a micro-enterprise is defined as an entity that, in at least one of the last two financial years, employed fewer than ten people and achieved an annual net turnover not exceeding two million euros or held total assets at year-end not exceeding this amount. A small enterprise, in turn, is an entity that, in at least one of the last two financial years, employed fewer than fifty people and achieved an annual net turnover not exceeding ten million euros or had total assets at year-end below this threshold.

This definition emphasizes businesses typically led by individual entrepreneurs with restricted growth trajectories but high levels of autonomy and owner involvement, reflecting the small scale of operations and limited financial and human resources, which are particularly relevant in the context of the present study on serial, portfolio, and novice entrepreneurs.

From a theoretical perspective, this study is grounded in trait theory, which seeks to explain entrepreneurship through relatively stable personality traits. While trait-based approaches were once criticized for their inconsistent results [9], more recent analyses and theoretical developments have demonstrated their utility, especially when applied to more specific entrepreneurial subgroups [30]. Traits such as risk propensity, need for achievement, self-efficacy, optimism, hope, resilience and openness to experience have been found to significantly affect entrepreneurial intentions and behaviour. This research combines Shane’s model with trait theory to explore how demographic and psychological variables interact across different types of entrepreneurs. In particular, the study focuses on:

- demographic factors such as: education, business experience, age, parental entrepreneurship, marital status and social status,
- psychological factors such as: extraversion, agreeableness, conscientiousness, emotional stability, openness to experience, risk propensity, hope, self-efficacy, optimism, resilience (resources of psychological capital), conformity versus nonconformity, and need for independence, autonomy, environmental mastery, purpose in life, self-acceptance.

### Demographic factors across entrepreneurial types

The role of demographic characteristics in explaining habitual entrepreneurship remains inconclusive in the literature. While some studies highlight clear patterns, others find no consistent relationships. One of the most consistently emphasized variables is prior business or professional experience. Research indicates that such experience enhances entrepreneurs’ ability to manage resources, understand markets, and develop effective business strategies [16,20,27]. Importantly, prior experience also fosters extensive networks, which are instrumental in opportunity recognition and execution, aligning with Shane’s model. However, the manifestation of experience differs across entrepreneurial types. Serial entrepreneurs tend to apply learning sequentially, refining their approach over successive ventures based on trial-and-error and reflective learning [20]. In contrast, portfolio entrepreneurs draw upon their accumulated knowledge in a parallel manner, leveraging their skills, contacts, and insights across multiple ventures at once. This requires greater cognitive and managerial flexibility, as well as advanced coordination skills [28].

Another critical demographic variable is education, which contributes to the development of strategic thinking, cognitive flexibility, and the ability to acquire and apply knowledge – traits that are essential in complex business environments [30]. Higher levels of education have been associated with improved opportunity recognition, more sophisticated decision-making, and access to broader networks and financial resources. Portfolio entrepreneurs often exhibit higher educational attainment, which is necessary for managing the complexity of multiple ventures [31]. In contrast, serial entrepreneurs may rely more on experiential learning and practical adaptation, rather than formal education [16]. Nevertheless, empirical findings remain mixed, with some studies failing to demonstrate a positive relationship between higher education and the propensity for repeat entrepreneurship [32,33].

Parental entrepreneurship is another influential factor. Growing up in a business-owning family can shape one’s attitudes toward risk, increase early exposure to business operations, and facilitate access to initial resources [34,35]. This background appears particularly relevant for portfolio entrepreneurs, who may benefit from family businesses, capital support, or informal mentorship – factors that enable them to sustain multiple ventures simultaneously [16,20]. Serial entrepreneurs, while also possibly influenced by entrepreneurial family backgrounds, may be more driven by internal motivation and individualistic aspirations, seeking new challenges across different ventures [17,28].

Age is frequently examined in relation to entrepreneurial pathways, though results remain inconclusive. Portfolio entrepreneurs are often older, leveraging accumulated experience, capital, and professional networks to manage multiple ventures effectively [3]. Serial entrepreneurs tend to be younger, demonstrating greater risk tolerance and flexibility, and are more likely to transition between ventures as they pursue new opportunities [20]. Despite these trends, age-related patterns in habitual entrepreneurship are not universally supported by empirical data.

Social and marital status also play a role in shaping entrepreneurial behaviour. Individuals with higher social standing or those who are married may benefit from greater emotional and financial stability, which can support risk-taking and business planning. Portfolio entrepreneurs may use this support to manage the complexities of running several ventures simultaneously. In contrast, serial entrepreneurs may prioritize autonomy and mobility, relying more on personal agency and flexibility [16].

### Psychological factors and trait theory across entrepreneurial types

From a psychological perspective, the present study draws on trait theory to analyze how enduring individual characteristics influence entrepreneurial behaviour. Meta-analyses conducted by Stewart and Roth [36], Zhao and Siebert [37], Rauch and Frese [38] and Brandstätter [39] reveal that traits such as agreeableness, conscientiousness, openness to experience, risk propensity, self-efficacy, and need for achievement are strongly associated with entrepreneurship. These traits influence how individuals perceive opportunities, tolerate ambiguity, and maintain commitment in the face of challenges. Self-efficacy, in particular, are critical for navigating entrepreneurial uncertainty and persisting through setbacks. Similarly, openness to experience and risk propensity foster the creativity and boldness required for innovation. Need for independence and nonconformity often drive individuals to pursue self-employment rather than traditional career paths [40–43].

Contemporary research expands trait theory’s application by incorporating affective and behavioural data from digital sources. For example, sentiment analysis of entrepreneurs’ Twitter content reveals patterns of optimism, stress, and opportunity perception, suggesting that real-time emotional states influence decision-making [44].

Moreover, the literature increasingly acknowledges the “darker side” of entrepreneurial psychology, where psychological contracts and power dynamics—especially in overly embedded contexts—may limit autonomy and suppress innovation [45]. This nuance is vital for understanding micro-entrepreneurship, where tight-knit social or family ties can be both a resource and a constraint.

In the context of habitual entrepreneurship, psychological traits likely manifest differently across types. Serial entrepreneurs may be characterized by a strong need for achievement, high risk appetite, and a tendency for exploration, driving them to move from one venture to the next. Research by [46] demonstrates that successful serial entrepreneurs often rely on intuitive pattern recognition derived from accumulated entrepreneurial experiences, enabling them to make faster and more confident decisions under uncertainty. This aligns closely with Shane’s emphasis on the iterative learning process and the refinement of opportunity recognition over successive ventures.

Portfolio entrepreneurs may demonstrate higher levels of self-efficacy, resilience, and strategic openness, allowing them to manage complex and simultaneous business operations. The inclusion of psychological capital – comprising hope, optimism, self-efficacy, and resilience – provides a modern and nuanced understanding of the psychological resources that support sustained entrepreneurial success [34]. Recent work by [24] adopts a resource redeployment perspective, showing that portfolio entrepreneurs actively shift financial, human, and social capital between their ventures to optimise performance. This dynamic capability not only supports resilience and diversification but also requires advanced cognitive flexibility and strategic risk assessment – traits strongly linked to psychological capital components such as self-efficacy and optimism. Integrating this perspective into Shane’s model highlights how demographic resources and psychological dispositions interact in real-time decision-making for concurrent business management.

In contrast, novice entrepreneurs may have less-developed psychological capital and less familiarity with the demands of entrepreneurship, making them an important comparative group for identifying differentiating traits among habitual entrepreneurs.

Few studies have systematically examined both demographic and psychological variables across all three groups – serial, portfolio, and novice entrepreneurs – using a unified conceptual framework. This gap is particularly evident in the context of micro-entrepreneurship, where entrepreneurial pathways are shaped not only by individual characteristics but also by limited resources, informal networks, and personal agency. To address these gaps, this study aims to empirically validate Shane’s model of entrepreneurship in the context of micro and small-scale. Based on these considerations, the following research questions were formulated:

1. *How do serial, portfolio, and novice entrepreneurs differ when it comes to key demographic characteristics such as:*
  1. *education,*
  2. *business experience,*
  3. *age,*
  4. *marital status,*
  5. *parental entrepreneurship,*
  6. *social status?*
2. *Do serial, portfolio, and novice entrepreneurs display distinct psychological profiles in terms of traits like:*
  1. *extraversion, agreeableness, conscientiousness, emotional stability, openness to experience,*
  2. *instrumental and stimulating risk-taking styles,*
  3. *hope, self-efficacy, optimism, resilience (resources of psychological capital),*
  4. *autonomy, environmental control, self-acceptance, life purpose, personal growth and positive relationships with others,*
  5. *conformity-nonconformity and heuristic-algorithmic behaviour?*

## Materials and methods

### Research sample and procedure

A total of 326 entrepreneurs participated in the study, comprising 110 serial entrepreneurs, 105 portfolio entrepreneurs, and 111 novice entrepreneurs (control group). The participants were recruited using a purposive sampling strategy, targeting individuals who met specific inclusion criteria related to entrepreneurial experience. The key criterion was ownership (minority or majority) of at least one independent business established before December 31, 2021. To ensure diversity, recruitment was conducted across multiple industry sectors (services, trade, manufacturing) and regions within Poland. However, no single sector or region was overrepresented. The sample also included entrepreneurs operating at different stages of business development (start-up, growth, maturity), allowing for variation in venture experience. The sampling strategy was designed to reflect the structure of the micro- and small-business environment in Poland, in which service enterprises predominate and habitual entrepreneurs often operate across sectors. This sampling approach supports the representativeness of the findings within the micro and small enterprise context, particularly in post-transition economies.

In the study group, the gender distribution was relatively balanced: 53.1% men, 44.5% women, while 1.5% refused to disclose their gender and 0.9% identified as another gender. Among novice entrepreneurs, women predominated (52.3%), whereas men were the majority among serial (52.7%) and portfolio entrepreneurs (60%). In terms of age, the largest cohort of participants was 35−44 years old (33.4%), followed by those aged 45−54 (26.1%) and 25−34 (25.2%). A small proportion (2.8%) were over 65. With respect to business size, micro-entrepreneurship (defined in this study as businesses employing fewer than 10 people and generating less than €2 million in annual turnover, following the European Commission, 2003 definition) was predominant in the research sample among serial entrepreneurs (55.5%) and novice entrepreneurs (61.3%). In contrast, portfolio entrepreneurs were more likely to operate small businesses (67.6%), often managing multiple companies simultaneously. The year of establishment of the businesses was evenly distributed. Among serial entrepreneurs, most ventures were founded before 2009 (29.1%) or between 2009−2016 (28.2%). A similar distribution was observed among portfolio entrepreneurs. In terms of industry, the majority of respondents operated in the services sector (62.3%). However, portfolio entrepreneurs were significantly more likely to own manufacturing companies (35.2%), compared to serial (12.7%) and novice entrepreneurs (16.2%). All participants were informed about the purpose and scope of the study and provided informed consent in accordance with the Declaration of Helsinki. Ethical approval was obtained from the Research Ethics Committee (Resolution No. 6/KEBN-UŁ/IV/2023–24) on 15 March 2024. The survey was administered online via LimeSurvey between 20 June and 18 July 2024. Respondents could proceed only after selecting “yes” to confirm their consent. Anonymity and confidentiality were assured throughout.

### Research tools

To examine the demographic and psychological characteristics distinguishing serial, portfolio, and novice entrepreneurs, the study employed the following standardized instruments. Each was selected based on theoretical alignment with Shane’s model of the entrepreneurial process and trait theory, as well as empirical relevance to entrepreneurial behaviour.

1. **Demographic Survey**. A structured survey questionnaire was used to assess key demographic variables that influence entrepreneurial behavior: age, education level, business experience, marital status, social status, and entrepreneurial family background. These variables function as structural filters, influencing access to resources, social capital, and exposure to opportunity, as proposed in Shane’s model.
2. **Ten Item Personality Inventory – Polish adaptation** **(TIPI-PL)** by Sorokowska et al. [47]. This tool measures the Big Five personality traits: extraversion, agreeableness, conscientiousness, emotional stability, and openness to experience, based on the Costa and McCrae model. Participants rated each item on a 7-point Likert scale. The measurement reliability of the TIPI-PL scales was evaluated using Cronbach’s coefficient α, which indicates internal consistency. All results were satisfactory, with the highest internal consistency coefficients found for Conscientiousness (0.85) and Agreeableness (0.84), followed by Extraversion (0.77), Emotional Stability (0.76), and Openness to Experience (0.71).
3. **Stimulating-Instrumental Risk Inventory (SIRI)** developed by Zaleśkiewicz [48]. SIRI distinguishes between two styles of risk-taking: instrumental (goal-oriented and analytical) and stimulating (sensation-seeking and affect-driven). These distinctions are particularly relevant in entrepreneurship, where calculated risk and opportunity-driven boldness may coexist or diverge across entrepreneurial types. The inventory contains 17 items on a 5-point Likert scale, with Cronbach’s α of 0.75 (stimulating) and 0.70 (instrumental).
4. **Psychological Capital Questionnaire (PCQ)** developed by Lipińska-Grobelny and Zwardoń-Kuchciak [49]. The PCQ measures hope, self-efficacy, resilience, and optimism – the four core dimensions of psychological capital (PsyCap). PsyCap has been shown to support entrepreneurial persistence, goal-setting, coping with failure, and opportunity exploitation. The 12-item tool uses a 6-point Likert scale, with high overall reliability (Cronbach’s α = 0.88; subscales 0.70–0.77).
5. **Scales of Psychological Well-Being (SPWB)** adapted by Krok [50]. This tool assesses six dimensions of psychological well-being: autonomy, environmental mastery, personal growth, positive relations, purpose in life, and self-acceptance. Several of these dimensions align with Shane’s model, especially regarding the role of personal agency and intrinsic motivation in entrepreneurial opportunity exploitation. The 42-item scale uses a 6-point Likert format, with internal consistency ranging from 0.52 to 0.69 depending on the subscale.
6. **Questionnaire of Creative Behaviour** (QCB III) developed by Bernacka, Popek, and Gierczyk [51]. The QCB III evaluates conformity vs. nonconformity and heuristic vs. algorithmic cognitive styles. These traits relate to entrepreneurial creativity, divergent thinking, and the ability to navigate uncertainty and generate innovative solutions. The tool includes 26 statements rated on a 5-point scale, with reliability coefficients between 0.74 and 0.78.

To minimize the risk of common method bias, the psychological instruments and demographic questionnaire were presented in a sequential order, reducing the likelihood of participants forming patterned responses across measures.

## Results

### Exploring demographic distinctions among serial, portfolio and novice entrepreneurs

All statistical analyses were conducted using IBM SPSS Statistics (version 29).

A Chi-square test of independence *(χ* ^2^), with effect size estimated by Cramer’s *V*, was used to assess whether the type of entrepreneurship is associated with selected demographic factors such as: education, age, parental entrepreneurship, marital and social status. In the case of differences between groups in the level of business experience, one-way analysis of variance (ANOVA) was used with effect size *eta²*.

The results of the Chi-square *(χ* ^2^) independence test showed that the level of education remained in a statistically significant relationship with the types of entrepreneurship (*χ²*(8) = 14.37; *p* = 0.05). A detailed analysis of the data presented in Table 1 revealed that the strength of the relationship between education and the types of entrepreneurship was fairly low (Cramer’s *V *= 0.15; *p *= 0.05). More than half (56.1%) of the respondents held a university degree. One in three entrepreneurs had completed their secondary education (33.7%). A relatively small group of the respondents declared primary, lower secondary, or basic vocational education, accounting for a total of 10.2%. All groups were predominantly composed of respondents with tertiary education. However, among the portfolio entrepreneurs, a significant proportion had completed their secondary education compared to the serial and novice entrepreneurs (43.8% versus 31.8% and 26.1%, respectively).

**Table 1:** Educational attainment of serial, portfolio, and novice entrepreneurs.

Educational attainment	Novice entrepreneurs		Serial entrepreneurs		Portfolio entrepreneurs		*χ*^*2*^ test *Cramer’s V*
**N**	**%**	**N**	**%**	**N**	**%**	
Primary	1	0.9	0	0.0	2	1.9	*χ*^2^ (8, *N *= 326) = 14,37 *p *= 0.05 Cramer’s *V *= 0.15 *p *= 0.05
Lower secondary	0	0.0	1	0.9	2	1.9	
Upper secondary – basic vocational education	9	8.1	12	10.9	6	5.7	
Upper secondary	29	26.1	35	31.8	46	43.8	
Tertiary education	72	64.9	62	56.4	49	46.7	
Total	111	100	110	100	105	100	

*Source: Compiled by the authors.*

Regarding differences in business experience among the serial, portfolio, and novice entrepreneurs, a one-way analysis of variance revealed no statistically significant differences (*F*(2,323) = 0.88; *p *> 0.05), (see Table 2). The average experience of the respondents was approximately 12 years (*M *= 11.93 ± 8.65). The serial entrepreneurs had slightly longer experience (*M *= 12.77 ± 9.06) compared to the portfolio group (*M *= 11.75 ± 8.40) and novices (*M* = 11.25 ± 8.48).

**Table 2:** Business experience (in years) of serial, portfolio, and novice entrepreneurs.

Types of entrepreneurs (N = 326)		*M*	*SD*	*F(2,323)*	*Significance*
Business experience	1. novices (N = 111)	11.25	8.48	0.88	0.42
	2. serial (N = 110)	12.77	9.06		
	3. portfolio (N = 105)	11.75	8.40		

*Explanation: M – mean, SD – standard deviation.*

*Source: Compiled by the authors.*

In the next step, the relationships between marital status and age and the types of entrepreneur were verified. The results of the Chi-square test showed that both age (*χ* ^2^(10)

= 7.74; *p* > 0.05) and marital status (*χ* ^2^(2) = 1.99; *p *> 0.05) were independent of the entrepreneurial types (see Tables 3-4). As shown in Table 3, the largest group of entrepreneurs were those aged between 35 and 44 (portfolio – 30.5%, serial – 38.2% and novice – 31.5%). Among the portfolio entrepreneurs, the 45–54 age category was equally well represented (28.6%), while for the serial entrepreneurs, the 25–34 age group (21.8%) and the 45–54 age group (24.5%) were significant. The novices had a higher proportion of 25–34 year olds (30.6%) than the portfolio and serial entrepreneurs.

**Table 3:** Age of serial, portfolio, and novice entrepreneurs.

Age (in years)	Novice entrepreneurs		Serial entrepreneurs		Portfolio entrepreneurs		*χ*^*2 *^ test
	N	%	N	%	N	%	
18-24	5	4.5	3	2.7	8	7.6	*χ*^2^ (10, *N* = 326) = 7.74 *p *= 0.65
25-34	34	30.6	24	21.8	24	22.9	
35-44	35	31.5	42	38.2	32	30.5	
45-54	28	25.2	27	24.5	30	28.6	
55-64	6	5.4	11	10.0	8	7.6	
65 and over	3	2.7	3	2.7	3	2.9	
Total	111	100	110	100	105	100	

*Source: Compiled by the authors.*

**Table 4:** Marital status of serial, portfolio, and novice entrepreneurs.

Marital status	Novice entrepreneurs		Serial entrepreneurs		Portfolio entrepreneurs		*χ*^*2*^ test
	N	%	N	%	N	%	
Married	75	67.6	83	75.5	78	74.3	*χ*^2^ (2, *N* = 326) = 1.99 *p *= 0.36
Unmarried	36	32.4	27	24.5	27	25.7	
Total	111	100	110	100	105	100	

*Source: Compiled by the authors.*

A majority of entrepreneurs were married (72.4%). The proportion of those in a marital relationship was slightly higher among the portfolio (74.3%) and serial (75.5%) entrepreneurs, compared to the novice entrepreneurs (67.6%). Unmarried individuals accounted for 27.6% of the total respondents, with the highest proportion observed among the novice entrepreneurs (32.4%), (see Table 4).

Further results of the *χ*^2^ independence test indicated that having an entrepreneurial parent had a statistically significant relationship with the types of entrepreneurship (*χ* ^2^(2) = 12.89; *p* < 0.05). However, the strength of this relationship was rather weak (Cramer’s *V *= 0.19; *p *< 0.05). A majority of the surveyed entrepreneurs reported that their parents had not been in business (72.4%). Significantly, this response was particularly dominant among the novice entrepreneurs (84.7%). In contrast, 27.6% confirmed that their parents had ran their own businesses. The percentage of such responses among the portfolio and serial entrepreneurs was higher compared to the novice entrepreneurs (35.2% and 32.7% vs. 15.3%, respectively), (see Table 5).

**Table 5:** Parental entrepreneurship among serial, portfolio and novice entrepreneurs.

Parent Entrepreneur	Novice entrepreneurs		Serial entrepreneurs		Portfolio entrepreneurs		*χ*^*2*^ test *V Cramer*
	N	%	N	%	N	%	
Yes	17	15.3	36	32.7	37	35.2	*χ*^2^ (2, *N* = 326) = 12.89 *p *= 0,002 *Cramer’s V *= 0.19 *p *= 0.002
Not	94	84.7	74	67.3	68	64.8	
Total	111	100	110	100	105	100	

*Source: Compiled by the authors.*

The list of demographic variables from Shane’s model concludes with self-reported social status. The results of the Chi-square test revealed that social position did not

correlate with the types of entrepreneurship (*χ* ^2^(6) = 7.96; *p* > 0.05), (see Table 6). Nearly half of the respondents considered themselves to belong to the group with average social status (45.1%). Among the portfolio entrepreneurs, this accounted for 51.4% of the respondents compared to 44.1% among the novices and 40% among the serial respondents. Almost 30% of the entrepreneurs were of the opinion that they enjoyed high social status, while 9.8% rated their social standing as low. In the group of serial entrepreneurs, the percentage of those who reported high social status was slightly higher compared to the portfolio and novice entrepreneurs (34.5% versus 26.7% and 25.2% respectively). This group also had the highest percentage of respondents who had difficulty assessing their position in society (19.1% vs. 12.4% and 17.1% respectively).

**Table 6:** Social status among serial, portfolio and novice entrepreneurs.

Social status	Novice entrepreneurs		Serial entrepreneurs		Portfolio entrepreneurs		*χ*^*2*^ test
**N**	**%**	**N**	**%**	**N**	**%**
High	28	25.2	38	34.5	28	26.7	*χ*^2^ (6, *N* = 326) = 7.96 *p *= 0.24
Average	49	44.1	44	40	54	51.4	
Low	15	13.5	7	6.4	10	9.5	
Difficult to say	19	17.1	21	19.1	13	12.4	
Total	111	100	110	100	105	100	

*Source: Compiled by the authors.*

### Exploring psychological distinctions among serial, portfolio and novice entrepreneurs

To test whether the type of entrepreneurship depends on the selected psychological factors from Shane’s model, specifically: (a) extraversion, conscientiousness, openness to experience, agreeableness, emotional stability, (b) risk propensity, (c) hope, self-efficacy, optimism, resilience, (d) autonomy, environmental control, self-acceptance, life purpose, personal growth, positive relations with others and (e) conformity-nonconformity, a series of one-way analyses of variance were performed, with *post hoc* tests verifying the significance of differences between the groups.

The result of these analyses indicated that the serial, portfolio, and novice entrepreneurs did not differ in a statistically significant way in their levels of extraversion (*F*(2,323) = 0.71; *p *> 0.05), conscientiousness (*F*(2,323) = 1.86; *p *> 0.05, openness to experience (*F*(2,323) = 0.96; *p *> 0.05), agreeableness (*F*(2,323) = 1.54; *p *> 0.05), or emotional stability (*F*(2,323) = 1.38; *p *> 0.05), (see Table 7). Notably, the results from the individual TIPI-PL scales revealed that all three groups of entrepreneurs were characterised by higher than average levels of extraversion, conscientiousness, openness to experience, agreeableness, and emotional stability.

**Table 7:** Extraversion, conscientiousness, openness to experience, agreeableness, and emotional stability(TIPI-PL) of serial, portfolio, and novice entrepreneurs.

Types of entrepreneurs (N = 326)		*M*	*SD*	*F(2,323)*	*Significance*
Extraversion	1. novice (N = 111)	9.47	3.24	0.71	0.49
	2. serial (N = 110)	9.86	3.02		
	3. portfolio (N = 105)	9.38	3.21		
Conscientiousness	1. novice (N = 111)	10.52	2.66	1.86	0.15
	2. serial (N = 110)	9.9	3.15		
	3. portfolio (N = 105)	10.57	2.73		
Openness to experience	1. novice (N = 111)	8.45	2.9	0.96	0.38
	2. serial (N = 110)	8.93	2.91		
	3. portfolio (N = 105)	8.91	2.98		
Agreeableness	1. novice (N = 111)	9.76	2.93	1.54	0.22
	2. serial (N = 110)	9.03	3.08		
	3. portfolio (N = 105)	9.39	3.25		
Emotional stability	1. novice (N = 111)	8.78	2.92	1.38	0.25
	2. serial (N = 110)	8.66	3.33		
	3. portfolio (N = 105)	9.31	2.83		

*Explanations: M – mean, SD – standard deviation.*

*Source: Compiled by the authors.*

In the next step, attention was focused on risk-taking styles. A one-way analysis of variance was again applied, with post hoc significance verification using the Bonferroni test.

As shown in Table 8, there were statistically significant differences between the serial, portfolio, and novice entrepreneurs in both stimulating risk (*F*(2,323) = 4.68; *p *< 0.05) and instrumental risk (*F*(2,323) = 6.3; *p *< 0.05). The effect size, measured by *eta²*, was close to moderate. The portfolio entrepreneurs exhibited the highest level of stimulating risk

**Table 8:** Risk appetite (SIRI) among serial, portfolio, and novice entrepreneurs.

Types of entrepreneurs (N = 326)		*M*	*SD*	*F(2,323)* *Eta²*	*Post hoc Bonferroni significance* *test*
Stimulating risk	4. novices (N = 111)	25.76	6.8	4.68 *Eta ² *= 0.03	0,01 Entrepreneurs 1-3; 2-3
	5. serial (N = 110)	25.97	6.48		
	6. portfolio (N = 105)	28.17	5.71		
Instrumental risk	1. novices (N = 111)	23.18	4.31	6.3 *Eta ² *= 0.04	0,002 Entrepreneurs 1-3; 2-3
	2. serial (N = 110)	23.93	4.51		
	3. portfolio (N = 105)	25.26	4.18		

*Explanations: M – mean, SD – standard deviation.*

*Source: Compiled by the authors.*

(*M* = 28,17 ± 5.71) compared to the serial entrepreneurs (*M* = 25,97 ± 6.48) and novices

(*M *= 25,76 ± 6.8). However, the mean score for the portfolio entrepreneurs remained below the mean value on the scale, suggesting that despite the observed differences, there is insufficient evidence to conclude that portfolio entrepreneurs are motivated by sensation-seeking, pleasurable stimulation, or excitement about managing a portfolio of companies. The same group of respondents (portfolio entrepreneurs) again scored the highest in instrumental risk (*M *= 25,26 ± 4.18) compared to the serial (*M *= 23.93 ± 4.51) and novice entrepreneurs (*M *= 23,18 ± 4,31). In this case, all three groups scored higher above the average value on the scale, but the portfolio entrepreneurs stood out as the leaders in adopting an instrumental risk-taking style. This suggests that when faced with uncertainty, portfolio entrepreneurs carefully analyse the balance between potential gains and losses, with their motivation to act being strengthened or weakened depending on situational factors, the availability of information, and the magnitude of possible outcomes, whether gains or losses.

Regarding the psychological capital variables (self-efficacy belief, hopefulness, resilience and optimism), the results of the one-way analysis of variance also confirmed significant differences between entrepreneurs. These differences were found in hopefulness (*F*(2,323) = 4.02; *p *< 0.05), and resilience (*F*(2,323) = 2.93; *p *= 0.05), (see Table 9). Although all groups demonstrated higher levels of these internal resources, the portfolio entrepreneurs, compared to the serial and novice entrepreneurs, exhibited the highest psychological capital. They also showed the strongest motivation, characterised by willpower and effective problem-solving abilities, as well as greater resilience enabling them to better cope with stress and adapt quickly to environmental changes.

**Table 9:** Psychological capital (KKaPsy) of serial, portfolio, and novice entrepreneurs.

Types of entrepreneurs (N = 326)		*M*	*SD*	*F(2,323)* *Eta²*	*Post hoc Bonferroni significance* *Test*
Self-efficacy belief	1. novices (N = 111)	12.48	2.81	1.89	0.15
	2. serial (N = 110)	12.66	2.87		
	3. portfolio (N = 105)	13.21	2.81		
Hopefulness	1. novices (N = 111)	12.92	2.44	4.02 *Eta ² *= 0.03	0.02 Entrepreneurs 1–3; 2–3
	2. serial (N = 110)	13.01	2.73		
	3. portfolio (N = 105)	13.83	2.56		
Resilience	1. novices (N = 111)	12.46	2.65	2.93 *Eta ² *= 0.02	0.05 Entrepreneurs 1–3
	2. serial (N = 110)	12.78	2.93		
	3. portfolio (N = 105)	13.38	2.81		
Optimism	1. novices (N = 111)	12.48	3.26	0.62	0.54
	2. serial (N = 110)	12.4	3.24		
	3. portfolio (N = 105)	12.85	3.02		

*Explanations: M – mean, SD – standard deviation.*

*Source: Compiled by the authors.*

The assessment of personality variables influencing entrepreneurial opportunities was completed by evaluating factors such as autonomy, self-acceptance, and the ability to control the environment (see Table 10), as well as conformity vs. non-conformity and heuristic vs. algorithmic behaviour (see Table 11).

**Table 10:** Autonomy, environmental control, self-acceptance, life purpose, personal growth and positive relationships with others (SPWB) among serial, portfolio, and novice entrepreneurs.

Types of entrepreneurs (N = 326)		*M*	*SD*	*F(2,323)*	*Significance*
Autonomy	1. novices (N = 111)	32.34	5.74	0.03	0.97
	2. serial (N = 110)	32.51	6.0		
	3. portfolio (N = 105)	32.47	5.64		
Environmental control	1. novices (N = 111)	31.77	6.01	1.52	0.22
	2. serial (N = 110)	31.51	6.23		
	3. portfolio (N = 105)	32.91	6.46		
Personal growth	1. novices (N = 111)	31.57	4.98	1.77	0.17
	2. serial (N = 110)	32.16	5.16		
	3. portfolio (N = 105)	32.86	4.93		
Positive relationships with others	1. novices (N = 111)	33.68	5.47	1.12	0.33
	2. serial (N = 110)	32.64	6.23		
	3. portfolio (N = 105)	33.54	5.04		
Life purpose	1. novices (N = 111)	32.99	5.16	0.68	0.50
	2. serial (N = 110)	32.31	6.52		
	3. portfolio (N = 105)	33.2	5.81		
Self-acceptance	1. novices (N = 111)	31.84	6.06	0.08	0.92
	2. serial (N = 110)	31.62	7.03		
	3. portfolio (N = 105)	31.99	6.42		

*Explanations: M – mean, SD – standard deviation.*

*Source: Compiled by the authors.*

**Table 11:** Conformity-nonconformity and heuristic-algorithmic behaviour (KANH-III) among serial, portfolio, and novice entrepreneurs.

Types of entrepreneurs (N = 326)		*M*	*SD*	*F(2,323)* *Eta ²*	*Post hoc Bonferroni significance* *test*
Conformity vs. non-conformity	1. novices (N = 111)	33.63	6.37	3.95 *Eta ² *= 0.03	0.02 Entrepreneurs 2-3
	2. serial (N = 110)	32.78	7.08		
	3. portfolio (N = 105)	35.41	7.44		
Heuristic vs. algorithmic behaviour	1. novices (N = 111)	32.84	6.59	1.91	0.14
	2. serial (N = 110)	32.75	6.42		
	3. portfolio (N = 105)	34.35	7.08		

*Explanations: M – mean, SD – standard deviation.*

*Source: Compiled by the authors.*

As in the previous cases, a one-way analysis of variance was performed, which revealed that the compared groups of entrepreneurs exhibited similar levels of autonomy (*F*(2,323) = 0.03; *p *> 0.05), self-acceptance (*F*(2,323) = 0.08; *p *> 0.05), ability to control the environment (*F*(2,323) = 1.52; *p *> 0.05), building positive relationships (*F*(2,323) = 1.12;

*p *> 0.05), achieving life purpose (*F*(2,323) = 0.68; *p *> 0.05), and actualising their potential (*F*(2,323) = 1.77; *p *> 0.05). The serial, portfolio, and novice entrepreneurs scored above the mean values on each of these scales (see Table 10).

The last to be considered were the differences between the entrepreneurs in their levels of conformity vs. nonconformity and heuristic vs. algorithmic behaviour. The data in

in Table 11 show that the serial, portfolio, and novice entrepreneurs differed in a statistically significant way in their level of non-conformity (*F*(2,323) = 3.95; *p *< 0.05), with the effect size of *eta²* close to moderate. The portfolio entrepreneurs exhibited the highest level of non-conformity, scoring above the mean value on the scale (*M *= 35.41 ± 7.44) compared to the serial entrepreneurs (*M *= 32.78 ± 7.08) and novices (*M *= 33.63 ± 6.37). This suggests that both portfolio entrepreneurs and novices are characterised by adaptive flexibility, tolerance, self-organisation, independence, courage, and high self-esteem. The lowest scores on this scale, below the mean value, were obtained by the serial entrepreneurs. As for heuristic vs. algorithmic behaviour, the differences between the groups were statistically insignificant (*F*(2,323) = 1.91; *p *> 0.05).

## Discussion

The aim of this research was to compare serial and portfolio entrepreneurs in terms of demographic and psychological characteristics. The findings revealed two demographic factors that were statistically significant. The first was education. Although all groups were predominantly composed of respondents with tertiary education, the portfolio entrepreneurs were more likely to hold a university degree than the serial entrepreneurs, who, along with the novices, more frequently had secondary education. This finding is intriguing as it suggests that a higher level of education does not necessarily correlate with a greater propensity to run habitual businesses simultaneously. According to Shane [2], individuals with higher education often have broader theoretical knowledge and analytical skills that may be useful in running a business. Additionally, these individuals may have better access to capital and networks. However, studies by Leonelli [33] and Westhead and Wright [16] did not confirm a direct link between educational attainment level and habitual entrepreneurship. Kurczewska and Mackiewicz [32] further argued that it is not the level of education but rather its diversity, e.g., graduating from different types of schools or in diverse fields of study, that is crucial. As such, the relationship between educational attainment and the likelihood of becoming a habitual entrepreneur is more nuanced than it initially might have seemed, with previous research offering no clear answers in this regard.

This interpretation resonates with the perspective of human and social capital, where education is not only a formal credential but also a means of building networks and enhancing access to resources [30]. At the same time, recent research suggests that the relationship between education and habitual entrepreneurship is far from linear: while higher education equips individuals with analytical skills, it does not unequivocally predict repeated entrepreneurial activity [32,33]. These findings indicate that the effect of education depends on how knowledge is combined with experience and embedded in entrepreneurial contexts.

Another demographic factor that significantly differentiates the types of entrepreneurs is whether they had entrepreneurial parents. The serial and portfolio entrepreneurs were notably more likely to report that their parents had been in business, compared to novice entrepreneurs. This factor may suggest that individuals raised in families with an entrepreneurial tradition may be more inclined to pursue habitual business ventures. Several factors could explain this influence. Firstly, early exposure to the business world can reduce the fear of entering the market, as children of entrepreneurs often grow up observing their parents managing a business. Secondly, access to capital may play a role, as entrepreneurial parents may provide their children with a source of funding for their own ventures, which offers a significant advantage when launching new ventures. Lastly, access to established business networks is another potential benefit. Entrepreneurs often build extensive business networks, which can be valuable for their children as they embark on their own entrepreneurial journeys. These results align with the literature on intergenerational entrepreneurship, which emphasizes that family background provides more than financial resources. Entrepreneurial parents transmit tacit knowledge, role models, and access to business networks that facilitate opportunity recognition and reduce perceived entry barriers [34,35]. This intergenerational transfer of entrepreneurial capital may help explain why portfolio entrepreneurs, in particular, are more likely to come from entrepreneurial families.

The study found no statistically significant differences between entrepreneurial types and demographic factors such as business experience, which contradicted the expectations that longer business experience would favour the emergence of serial or portfolio entrepreneurs. Additionally, age did not appear to influence entrepreneurial preferences either, as the respondents across different age groups exhibited similar tendencies towards various entrepreneurial types. Similarly, marital status – whether the respondents were married or not – had no discernible effect on their entrepreneurial type preferences. Furthermore, self-perceived social standing did not show a significant relationship with the type of entrepreneurship pursued. These non-significant findings warrant further reflection. Prior research has reported mixed evidence on the role of age and experience in differentiating habitual from novice entrepreneurs. While some studies suggest that greater business experience or older age should facilitate repeated entrepreneurial activity [20,52], other findings indicate that these factors are not consistently predictive once psychological traits and contextual resources are taken into account [32,38]. The lack of effect in this study may reflect the relative homogeneity of the sample in terms of age and career stage, as well as the possibility that demographic characteristics are less decisive than psychological resources and family background in explaining entrepreneurial typologies. This interpretation reinforces the view that entrepreneurship is shaped by a complex interplay of individual and contextual factors, rather than by demographic characteristics alone.

With regard to the psychological factors highlighted in Shane’s model, differences between the portfolio and serial entrepreneurs were confirmed in the area of risk-taking, psychological capital, and non-conformity. The willingness to take risks in Shane’s model is one of the most important factors influencing entrepreneurial decisions and the ability to identify and pursue opportunities. The analyses presented here indicated that the portfolio entrepreneurs were characterised by higher levels of both stimulus and instrumental risk compared to the serial entrepreneurs and novices. The serial entrepreneurs ranked second in terms of instrumental risk levels. The elevated levels of instrumental risk in the portfolio entrepreneurs may be attributed to the excitement and variety associated with managing multiple companies. Nevertheless, it is important to note that even though the portfolio entrepreneurs scored highest on this risk scale, their scores were still lower than the mean value on the scale. In contrast, the higher levels of instrumental risk in the portfolio entrepreneurs may indicate that their decisions to start more companies are well thought out, based on thorough analysis and effective risk management. This pattern is consistent with meta-analytical findings showing that entrepreneurs, compared to managers, tend to display stronger risk propensity, which facilitates opportunity exploitation and persistence in entrepreneurial careers [36,37]. Importantly, the higher instrumental rather than stimulating risk orientation among portfolio entrepreneurs suggests calculated and strategic risk-taking rather than impulsivity.

Furthermore, portfolio entrepreneurs demonstrated significantly higher levels of two key components of psychological capital – hope and resilience – in comparison to serial and novice entrepreneurs. While all three groups scored above the population mean on psychological capital, portfolio entrepreneurs consistently showed the highest ratings, suggesting a particularly strong psychological resource base. This finding may reflect the unique demands and cognitive load associated with managing multiple ventures simultaneously. Portfolio entrepreneurship requires a high degree of strategic flexibility, adaptability, and stress tolerance, which likely both attracts individuals with high PsyCap and fosters its further development. Specifically, resilience – the ability to recover from setbacks and sustain effort under pressure – appears particularly critical in the portfolio context, where entrepreneurs must juggle the distinct risks, goals, and operational challenges of each venture. As previous research suggests, these individuals are more frequently exposed to complex, high-stakes decision-making environments [16], which may promote the reinforcement or selection of resilient dispositions. Similarly, hope conceptualized as a motivational state that involves both goal-directed energy (agency) and planning to meet goals (pathways thinking) – plays a central role in entrepreneurial persistence and strategic vision [53]. For portfolio entrepreneurs, hope may be especially salient in managing the interconnected goals and trade-offs across several businesses. Their higher scores on hopefulness could reflect a more goal-structured and optimistic outlook, necessary for pursuing long-term gains despite short-term volatility. These findings are consistent with psychological research that highlights resilience and hope as critical personal resources. Meta-analyses confirm that psychological capital is positively associated with entrepreneurial persistence and success [38,39] In particular, resilience enables entrepreneurs to cope with adversity, while hope fosters goal-directed energy and planning [40]Such resources appear to be especially valuable in the more complex context of portfolio entrepreneurship.

Overall, these patterns may be explained by a combination of selection effects (individuals high in PsyCap self-selecting into portfolio entrepreneurship) and development effects (the entrepreneurial experience itself enhancing psychological capital over time). The interaction of these forces supports Shane’s model of entrepreneurship, which posits that entrepreneurial outcomes result from the dynamic interplay between personal characteristics and environmental demands.

An important implication of these findings is the potential for interaction effects between demographic and psychological factors. For instance, entrepreneurs with parental business background may be better positioned to translate resilience and hope into sustained entrepreneurial activity, combining inherited networks with personal psychological strengths. Similarly, education may amplify the benefits of psychological capital by enabling entrepreneurs to apply resilience and risk assessment more strategically. Future studies should therefore test integrative models that move beyond isolated analyses of demographic and psychological determinants.

In addition, the portfolio entrepreneurs showed higher levels of non-conformity, suggesting greater independence, a willingness to challenge the status quo, and a commitment

to following their own paths. They appear to be more flexible, tolerant, and courageous in their actions. It can also be inferred that these traits align with those indicated by Shane: self-confidence, intuition, self-efficacy beliefs, and the ability to generalise. This result resonates with studies showing that non-conformity and openness to experience are strongly linked to creativity and opportunity recognition in entrepreneurship [38–39] By challenging established norms and exploring unconventional strategies, portfolio entrepreneurs may create the flexibility necessary to operate and innovate across multiple ventures simultaneously.

Traits common to all types of entrepreneurs were found to include higher-than-average levels of extraversion, conscientiousness, openness to experience, agreeableness, autonomy, sense of control, and emotional stability. Although no significant differences were found between the groups regarding these traits, it is worth noting that all three groups exhibited higher-than-average levels. This suggests that these personality traits may be generally desirable in the context of running a business, regardless of the type of entrepreneur.

The study underscores the nuanced differences between serial and portfolio entrepreneurs. Serial entrepreneurs rely more on experiential learning, leveraging sequential ventures for refinement and growth, while portfolio entrepreneurs emphasize diversification, resilience, and complex management strategies. Psychological capital and exposure to entrepreneurial environments emerge as critical factors in shaping entrepreneurial pathways. Future research could delve deeper into the interplay of these factors, offering tailored strategies for supporting different types of entrepreneurs.

While Shane’s model has been widely applied to nascent and growth-oriented entrepreneurship, relatively little research has examined its applicability to micro- and small-scale ventures—particularly those led by habitual entrepreneurs. This study addresses three specific gaps in the current understanding.

First, prior work has largely overlooked how the resource constraints and market embeddedness typical of micro-enterprises influence the opportunity recognition–exploitation process described in Shane’s framework. Our findings show that these constraints shape both the cognitive and behavioural mechanisms entrepreneurs use, suggesting the need for a refined model that incorporates context-dependent resource mobilisation strategies.

Second, empirical applications of Shane’s model rarely distinguish between serial, portfolio, and novice entrepreneurs, even though these groups differ in their experience-based heuristics, network structures, and risk–return profiles. By integrating these distinctions into the analysis, we contribute a more nuanced understanding of how the model operates across different entrepreneurial career paths.

Third, the study identifies interaction effects between demographic and psychological factors—an element not explicitly addressed in the original model—that better explain entrepreneurial persistence and typology formation. This extension adds a socio-psychological layer to Shane’s economic–cognitive emphasis, thereby enhancing its explanatory power in diverse entrepreneurial contexts.

These observations resonate with longitudinal evidence that entrepreneurial identity and self-efficacy can evolve through successive venture experiences [54,55], and that psychological resource-based approaches offer a dynamic perspective on persistence beyond fixed trait models [56]. Collectively, these contributions not only validate Shane’s framework in an underexamined setting but also refine it to better capture the realities of micro- and small-scale entrepreneurship.

## Conclusions

Based on the conducted analyses, it was possible to draw conclusions regarding the diversity of demographic and psychological characteristics among serial, portfolio and novice entrepreneurs:

1. Demographic Factors:

- Educational attainment: Portfolio entrepreneurs were more likely to hold university degrees than their serial or novice counterparts. However, the diversity of educational backgrounds may be more influential than the level alone;
- Entrepreneurial family background: Serial and portfolio entrepreneurs more often came from entrepreneurial families, indicating that early exposure to business, access to entrepreneurial networks, and lower psychological entry barriers may foster habitual entrepreneurship.

1. Psychological Factors:

- Hope and resilience: Portfolio entrepreneurs showed the highest levels of hope and resilience – key psychological resources for successfully managing multiple ventures simultaneously;
- Selection vs. development effects: These traits may reflect both selection effects (individuals with higher psychological capital gravitate toward portfolio entrepreneurship) and developmental effects (the experience of managing multiple businesses may further enhance these traits).
- Theoretical alignment: The findings align with Shane’s model, emphasizing the interaction between individual characteristics and environmental demands in shaping entrepreneurial behaviour.
- Risk-taking and non-conformity: Portfolio entrepreneurs exhibited higher levels of both stimulating and instrumental risk-taking, along with greater non-conformity – traits linked to independence, innovation, and adaptive thinking, all crucial for entrepreneurial effectiveness.
- Psychological trait profile across groups: All entrepreneurial groups scored above the average on traits such as extraversion, conscientiousness, openness to experience, agreeableness, emotional stability, autonomy, and internal locus of control. These characteristics appear to be fundamental assets across all types of entrepreneurs.
- Habitual entrepreneurs are not homogeneous: The findings support the view previously emphasized by Westhead and Wright, that habitual entrepreneurs (serial and portfolio) are not a uniform group and should be studied as distinct categories.

Importantly, the study moves beyond simply validating Shane’s model. Three novel contributions can be highlighted:

- Contextual extension: By focusing on micro and small firms, the research demonstrates how resource constraints and market embeddedness influence the opportunity recognition–exploitation process, refining Shane’s framework for contexts often overlooked.
- Differentiation within habitual entrepreneurs: By clearly distinguishing between serial and portfolio entrepreneurs, the study uncovers divergent psychological profiles and strategic logics that are masked when habitual entrepreneurs are treated as a single group.
- Combined influence of factors: The findings highlight how demographic background (e.g., entrepreneurial family tradition, educational diversity) and psychological resources (e.g., resilience, hope) jointly shape entrepreneurial persistence and typology formation.

This adds a socio-psychological dimension to Shane’s originally economic–cognitive emphasis. Together, these contributions extend the explanatory scope of Shane’s model and provide a more fine-grained theoretical account of entrepreneurial behaviour across different career paths.

### Practical implications

The differentiation between novice, serial, and portfolio entrepreneurs has direct implications for the design of educational programmes and policy interventions. For novices, early-stage training should focus on building psychological capital (self-efficacy, resilience, optimism) alongside foundational business skills, compensating for limited experiential knowledge. Serial entrepreneurs, in turn, may benefit from modules on strategic renewal to avoid over-reliance on heuristics from prior ventures, while portfolio entrepreneurs require advanced training in multi-venture resource orchestration and coordination mechanisms. Understanding these differences is crucial for developing effective support programmes that address the specific challenges of each group—for instance, providing novices with mentorship networks, serials with innovation incentives, and portfolios with coordination platforms. Such initiatives should also include access to funding, mentoring, and networking opportunities, particularly important for habitual entrepreneurs. Moreover, research on this group contributes to a better understanding of the factors influencing economic development, offering valuable insights for policies that foster firm survival, growth, and job creation.

### Limitations of the study

The research findings presented here also have certain limitations. Firstly, the study was conducted on a population smaller than the full scope of the SME sector. Researchers often use the term SME to assess the health of the businesses that represent it. The exclusion of medium-sized companies from participation in the research was intentional, given their significant differences from micro and small enterprises regarding the role played within them by the entrepreneur. In Shane’s model, the focus on demographic and psychological factors highlights the active and fully causal role of entrepreneurs, which is much less common in medium-sized companies.

Secondly, while Shane’s model provides a valuable framework for analysing entrepreneurial behaviour, its original formulation was developed primarily in the context of nascent entrepreneurs. Applying this model to experienced entrepreneurs has certain limitations. Experienced entrepreneurs often rely on accumulated knowledge, networks, and prior business experience, which can alter the relevance of some demographic and psychological variables central to Shane’s framework. As a result, some aspects of the model may oversimplify or fail to capture the more complex, iterative, and path-dependent decision-making processes of seasoned entrepreneurs. This limitation should be taken into account when interpreting the findings, as the explanatory power of Shane’s model may differ depending on the entrepreneurial experience of respondents. The third limitation is the in-depth nature of the study, which may not be directly comparable to similar research conducted worldwide. This stems from the authors’ determination to describe demographic and psychological factors in as much detail as possible, utilizing interdisciplinary methods grounded in the current state of knowledge in the discipline of management and in the discipline of psychology. The absence of such studies in both the domestic and international literature hinders the ability to make direct comparisons. Nonetheless, this situation draws attention to the research gap in this area and gives rise to a recommendation to expand the geographical scope of future research encompassing both demographic and psychological factors.

Finally, another limitation identified by the authors pertains to the developmental phase of research on the topic, which does not yet provide clear guidelines for support policies aimed at micro and small entrepreneurs. The authors are aware of the need to increase the applicability of their research findings. In the current turbulent environment, it is essential to differentiate between support tools for serial and portfolio entrepreneurs compared to novice entrepreneurs. Increasing the practical value of this research is a primary objective for the authors in their further research work, and this article serves as the first part of that endeavour.

## Supporting information

S1 File Database. (XLSX)
